# Evaluation of Movement Restriction Zone Sizes in Controlling Classical Swine Fever Outbreaks

**DOI:** 10.3389/fvets.2016.00124

**Published:** 2017-01-10

**Authors:** Shankar Yadav, Nicole Olynk Widmar, Donald C. Lay, Candace Croney, Hsin-Yi Weng

**Affiliations:** ^1^Department of Comparative Pathobiology, Purdue University, West Lafayette, IN, USA; ^2^Department of Agricultural Economics, Purdue University, West Lafayette, IN, USA; ^3^USDA-ARS Livestock Behavior Research Unit, West Lafayette, IN, USA; ^4^Purdue University Center for Animal Welfare Science, West Lafayette, IN, USA

**Keywords:** classical swine fever, CSF, movement restriction, outbreak control, foreign animal diseases, pig, swine

## Abstract

The objective of this study was to compare the impacts of movement restriction zone sizes of 3, 5, 9, and 11 km with that of 7 km (the recommended zone size in the United States) in controlling a classical swine fever (CSF) outbreak. In addition to zone size, different compliance assumptions and outbreak types (single site and multiple site) were incorporated in the study. Three assumptions of compliance level were simulated: baseline, baseline ± 10%, and baseline ± 15%. The compliance level was held constant across all zone sizes in the baseline simulation. In the baseline ± 10% and baseline ± 15% simulations, the compliance level was increased for 3 and 5 km and decreased for 9 and 11 km from the baseline by the indicated percentages. The compliance level remained constant in all simulations for the 7-km zone size. Four single-site (i.e., with one index premises at the onset of outbreak) and four multiple-site (i.e., with more than one index premises at the onset of outbreak) CSF outbreak scenarios in Indiana were simulated incorporating various zone sizes and compliance assumptions using a stochastic between-premises disease spread model to estimate epidemic duration, percentage of infected, and preemptively culled swine premises. Furthermore, a risk assessment model that incorporated the results from the disease spread model was developed to estimate the number of swine premises under movement restrictions that would experience animal welfare outcomes of overcrowding or feed interruption during a CSF outbreak in Indiana. Compared with the 7-km zone size, the 3-km zone size resulted in a longer median epidemic duration, larger percentages of infected premises, and preemptively culled premises (*P*’s < 0.001) across all compliance assumptions and outbreak types. With the assumption of a higher compliance level, the 5-km zone size significantly (*P* < 0.001) reduced the epidemic duration and percentage of swine premises that would experience animal welfare outcomes in both outbreak types, whereas assumption of a lower compliance level for 9- and 11-km zone sizes significantly (*P* < 0.001) increased the epidemic duration and percentage of swine premises with animal welfare outcomes compared with the 7-km zone size. The magnitude of impact due to a zone size varied across the outbreak types (single site and multiple site). Overall, the 7-km zone size was found to be most effective in controlling CSF outbreaks, whereas the 5-km zone size was comparable to the 7-km zone size in some circumstances.

## Introduction

Classical swine fever (CSF) is one of the most economically devastating diseases affecting the swine industry and is currently listed as a Class A foreign animal disease in the United States (1). CSF was first identified in the United States in 1833 and was eradicated in 1976 (2). Currently, CSF is present in Asia, Eastern Europe, and South America. CSF emerged in several disease-free countries and caused severe consequences in the past decades (3). For example, in 1997–1998, severe CSF outbreaks were reported from the Netherlands, Germany, Italy, and Spain. In the Netherlands alone, 11 million pigs were culled and $2.3 billion were spent during the outbreak control (4). In 2001–2002, CSF outbreaks in Catalonia, Spain, led to slaughter of 0.29 million pigs (5).

**Figure 1:**
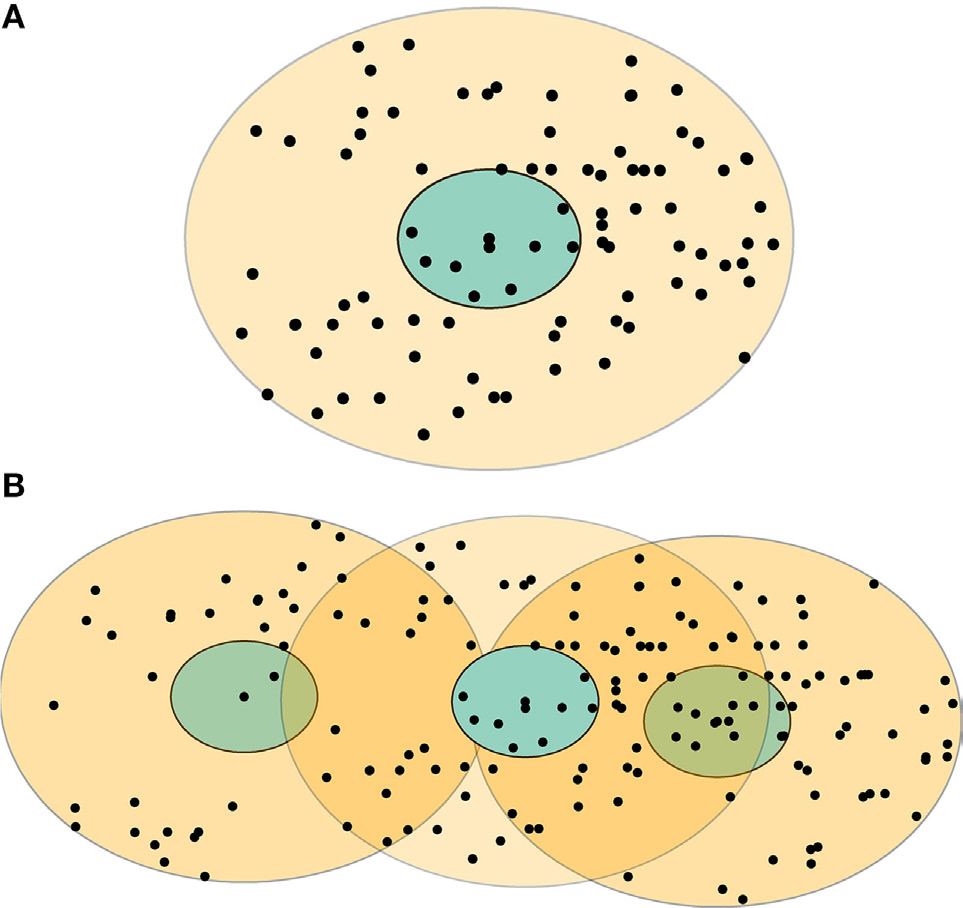
**Examples of the control zones for two types of classical swine fever outbreaks: (A) single-site and (B) multiple-site outbreaks**. The blue area represents an infected zone, and the light yellow area represents a movement restriction zone. The black dots represent individual swine premises. Some swine premises might be included in more than one movement restriction zones (the dark yellow areas) in a multiple-site outbreak.

Movement restriction is the most essential strategy for CSF outbreak control (6–8). In the United States, movement restrictions will be implemented and enforced on infected, suspected, and contact swine premises in the infected zone (a 3-km radius surrounding the infected premises) and on the uninfected swine premises in the movement restriction zone (7 km away from the perimeter of the infected zone; see Figure 1A) during a CSF outbreak (9). In the movement restriction zone, movement restrictions will be enforced until 28 days after the disinfection of the last infected premises (9). During this period, the unauthorized movements of pigs, vehicles, and swine farm workers beyond the designated movement restriction areas will be prohibited (9). For the effective implementation of movement restriction, selection of suitable movement restriction zone size could be vital, and it might necessitate consideration of various aspects, such as the initial outbreak types (single-index premises versus multiple-index premises), compliance levels, available resources, and the consequences (e.g., adverse animal welfare outcomes) of movement restriction.

A high level of compliance with movement restriction is essential for effective management of CSF outbreaks (6). However, several factors might contribute to the maintenance of a high compliance level with movement restrictions. For example, compliance level might be dependent on the movement restriction zone size, the progress of an ongoing outbreak, or the initial outbreak types. A smaller movement restriction zone size would require less enforcement to maintain the same level of compliance. A lower compliance with movement restrictions might be observed during the early stages of an epidemic due to lack of proper communication among stakeholders. On the other hand, a prolonged epidemic might result in a lower compliance level toward the later stage of an outbreak. In the past, a maximum of 85% compliance with movement restriction was estimated during the 1997–1998 CSF outbreak in the Netherlands (8).

Despite its crucial role in CSF outbreak control, various complications might arise due to implementation of movement restriction. For example, US swine herds are operated utilizing the maximum available spaces, and movement restriction might give rise to overcrowding in the swine herds (10, 11). Also, movement restriction on feed carrying vehicles might cause feed supply interruptions in swine herds (10, 12). These consequences could induce animal welfare problems among the pigs (10, 11). In overcrowded swine herds, pigs cannot express their natural behaviors (e.g., exploration or chewing an object), which might result in tail-biting, ear-chewing, aggression, fighting, and wounds (13, 14). During a CSF outbreak, the extent and magnitude of such animal welfare outcomes in the affected areas might be dependent on the movement restriction zone size. Historical CSF control programs encountered insufficient resources for effective management due to the large number of depopulated swine premises that resulted from infection, preemptive culling, and animal welfare problems, which complicated the outbreak management and extended the outbreak duration (15, 16).

Compared with other CSF control strategies (e.g., preemptive culling and vaccination), movement restriction has been reported to be the most effective in controlling CSF outbreaks (6). However, only limited studies directly evaluated the impacts of movement restriction zone sizes on different CSF outbreak-related outcomes. During the 1997–1998 CSF outbreak in the Netherlands, movement restrictions were inconsistently imposed, and the zone size was extended while the epidemic was at its peak (8). Such inconsistencies in the implementation of movement restriction zone sizes could negatively affect outbreak control efforts. Most of the CSF simulation studies have incorporated a fixed movement restriction zone size (17–20). Choices of movement restriction zone size in previous studies were different than the recommended movement restriction zone size in the United States. Moreover, none of the previous studies evaluated the impacts of movement restriction zone sizes on animal welfare concerns of pigs under movement restriction. Therefore, we conducted this study in the context of the Indiana swine industry to evaluate the impacts of the selected movement restriction zone sizes (3, 5, 9, and 11 km) on CSF outbreak-related outcomes [e.g., epidemic duration (ED), percentage of infected premises, preemptively culled premises, and premises affected by adverse animal welfare outcomes] compared with 7-km zone size, which is the recommended zone size in the US CSF outbreak emergency response plan (9).

Indiana is the top fifth pork producing state in the United States. In 2012, there were 8,631 registered swine premises with herd size ranging from 1 to 20,000. The Indiana swine industry imported 2.8 million and exported 1.5 million live pigs in 2012. The majority of the swine premises were finisher operations (59%), followed by farrow-to-finish (36%) and nursery (5%) operations.

## Materials and Methods

In this study, the Indiana State Swine Premises Identification Database (USAHERDS) of 2012 was used. The USAHERDS contained information on individual swine premises and live pig import and export activities. Each individual swine premise was identified by a unique swine premises identification number, geolocation (latitude and longitude), herd size, and operation type. In a previous study, we developed risk metrics to identify the most-likely CSF outbreak scenarios in Indiana (21). We randomly selected 4 (of 19) single-site (i.e., 1 initial outbreak site) and 4 (of 15) multiple-site (i.e., more than 1 initial outbreak sites) CSF outbreak scenarios for outbreak simulations in this study (21). The four multiple-site outbreak scenarios had 26, 20, 17, and 4 initial outbreak sites (i.e., index premises).

ArcGIS (version 10.3, ESRI, Redlands, CA, USA) was used to map the infected zone (3-km radius) and movement restriction zones with the sizes of 3, 5, 7, 9, and 11 km (away from the perimeter of the infected zone) for each of the index premises (*n* = 71) in the eight CSF outbreak scenarios. An example of infected and movement restriction zones and the distribution of swine premises within these control zones in the single-site and multiple-site CSF outbreak types are present in Figures 1A,B, respectively.

A two-step modeling approach was employed in the study. First, CSF spread was simulated using the North American Animal Disease Spread Model (NAADSM) to estimate the impacts of movement restriction zone sizes on ED, percentage of infected, and preemptively culled premises. Second, a novel animal welfare risk assessment model was developed to estimate the percentage of swine premises that would experience animal welfare outcomes of overcrowding and feed interruption under different movement restriction zone sizes.

### Simulation of CSF Spread

North American Animal Disease Spread Model software PC version 4.0.13 was used to simulate CSF spread in Indiana. NAADSM is an open-source software for simulations of infectious disease outbreaks and their control measures. The NAADSM integrates stochastic, temporal, and spatial modeling characteristics (22). For CSF spread simulation, two main data sources were used: empirical data (i.e., USAHERDS) and published literature. Geolocation (latitude and longitude), herd size, and operation type of Indiana swine premises in 2012 were used to simulate the spatial spread of CSF among swine premises. Data on virus transmission were obtained from the published literature (Table 1).

**Table 1:** **Premises-level model input parameters and probability distributions used for the simulation of classical swine fever spread in Indiana, United States**.

Parameter	Probability distribution	Reference
Latent period (days)	Poisson (4)	(6)
Subclinical period (days)	Poisson (6)	(23)
Clinical period (days)	Poisson (21)	(20)
Mean direct contact rate (recipient premises/premises/day)	Poisson (0.186)	(24)
Probability of infection transfer after direct contact	0.277	(25)
Mean indirect contact rate (recipient premises/premises/day)	Poisson (0.3)	(26)
Probability of infection transfer after indirect contact	0.048	(25)
Maximum distance for contact between premises	Triangle (1, 60, 120)	USAHERDS^a^
Between-premises distance matrix	Computed from data	USAHERDS^a^

*^a^2012 Indiana Swine Premises Identification Database*.

All Indiana swine premises (*N* = 8,631) registered in the 2012 USAHERDS were included in the model simulations. At the start of simulations (day 0), all swine premises were labeled as susceptible status except for the index premises, which were labeled as latently infected status. The latently infected premises were allowed to transit to subclinical and clinical disease status. The duration of different stages of CSF infection (i.e., latent, subclinical, and clinical) and the corresponding probability distributions were obtained from published studies (6, 20, 23) (Table 1). The transmission of viruses from the infected premises to susceptible premises was allowed through three modes: direct contact, indirect contact, and local spread. A direct contact was defined as the spread of CSF from infected premises to susceptible premises through shipment of live pigs, and indirect contact was through movement of vehicles, people, and equipment. A local spread was defined as the spread of CSF from infected premises to susceptible premises within a 1-km radius (22, 27, 28). For the spread of viruses *via* direct or indirect contact, contact rates, probability of infection transfer, and distance distribution of recipient premises were specified. The input parameters for contact rates and probability of disease transfer were adopted from published studies (20, 24, 26). The NAADSM used the geolocation of swine premises in Indiana to compute the between-premises distance matrix. The probability of disease transfer was modeled to gradually decrease as the distance from an infected premises increased (22, 28). Three CSF outbreak control strategies to be implemented in the United States (1) were incorporated in the model simultaneously: movement restriction; vaccination; and depopulation of vaccinated, infected, and contact premises. Vaccination (live attenuated) was implemented in the model for all swine premises in the infected zone (3-km radius) (1, 9, 29). The depopulation capacity was modeled to gradually increase from 0 to 10 premises/day by day 7 and 15 premises/day by 15 days onward (24). Additional details on the model parameters and model descriptions can be found elsewhere (21, 22, 28).

Three different compliance assumptions were simulated: baseline, baseline ± 10%, and baseline ± 15%. Movement restriction zone sizes were dichotomized into two groups: small (3 and 5 km) and large (9 and 11 km). In the baseline, compliance level was held constant across all zone sizes. In the baseline ± 10%, the compliance level was increased by 10% from the baseline for the small zone size group and decreased by 10% for the large group. In the baseline ± 15%, the compliance level was increased by 15% from the baseline for the small group and decreased by 15% for the large group. The compliance level for 7-km zone size remained constant in all simulations. The compliance level was modeled by altering direct contact (e.g., movement of pigs) and indirect contact (e.g., movement of vehicles and people) rates in the NAADSM. For example, a direct contact rate reduced to 25% reflected a compliance level of no movement of pigs of 75%, and an indirect contact rate reduced to 35% reflected a compliance level of no movement of people and vehicles of 65%. The baseline compliance levels, modeled as direct and indirect contact rates, by different outbreak types are summarized in Table 2. We incorporated slightly lower compliance levels for multiple-site outbreak type compared with single-site outbreak assuming that a larger number of swine premises and broader geographical areas in the multiple-site outbreak type would reduce compliance due to a greater demand of resources for enforcement. Indirect contacts were assumed to have a lower compliance than direct contacts due to potentially low adherence of people and vehicles with movement restriction. All simulations were run separately for each of the five movement restriction zone sizes, two outbreak types (with eight outbreak scenarios), and three compliance levels with 500 iterations each. Previous studies have demonstrated that this number of iterations was sufficient to generate reliable output estimates (22, 24).

**Table 2:** **Baseline compliance level with movement restrictions expressed as the percent reduction in the direct and indirect contact rates used in the classical swine fever spread model by two types of outbreaks (i.e., single-site outbreak and multiple-site outbreak)**.

Day	Compliance levels			
	Single-site outbreak		Multiple-site outbreak	
	Direct contact rate	Indirect contact rate	Direct contact rate	Indirect contact rate
1	0	0	0	0
7	75%	65%	70%	65%
15	75%	65%	70%	65%
30	75%	60%	70%	60%
60	65%	60%	60%	60%

### Animal Welfare Risk Assessment Model

A novel stochastic risk assessment model was developed to estimate the percentage of swine premises that would experience adverse animal welfare outcomes due to movement restrictions in the context of the Indiana swine industry. The animal welfare outcomes investigated were overcrowding or feed interruption on the finisher swine operations, which were the major swine operations in Indiana in 2012. The unit for the model simulation was swine premises and simulations proceeded by a time step of one day. The probability distribution of input model parameters such as the unique number of swine premises that would fall under movement restrictions during an outbreak was estimated using the 2012 Indiana swine premises data. The estimation of probability distribution for ED was described in this section, whereas the time elapsed between the onset of an outbreak and the emergence of animal welfare concerns (TWC) was adopted from our previous study (10). Details on the input model parameters and their probability distributions are summarized in Table 3 and are briefly described below.

**Table 3:** **Input model parameters and probability distributions (estimated using the 2012 Indiana swine premises data) for animal welfare risk assessment models by movement restriction (MR) zone size**.

Parameters	Movement restriction zone sizes				
	3 km	5 km	7 km	9 km	11 km
Epidemic duration (ED)					
Single site: ED low	Triangle (0, 30, 130)	Triangle (0, 30, 130)	Triangle (0, 30, 130)	Triangle (0, 30, 130)	Triangle (0, 30, 130)
Single site: ED high	Triangle (210, 290, 450)	Triangle (170, 250, 470)	Triangle (170, 260, 490)	Triangle (130, 250, 550)	Triangle (130, 250, 600)
Single site: Prob_ED_low	Bernoulli (0.27)	Bernoulli (0.28)	Bernoulli (0.28)	Bernoulli (0.3)	Bernoulli (0.3)
Multiple site	Triangle (229, 258, 316)	Triangle (186, 222, 313)	Triangle (171, 214, 315)	Triangle (164, 214, 337)	Triangle (161, 215, 336)
Time to animal welfare concern (TWC)					
Single site	Triangle (2, 62, 165)	Triangle (2, 62, 165)	Triangle (2, 62, 165)	Triangle (2, 62, 165)	Triangle (2, 62, 165)
Multiple site	Triangle (2, 61, 173)	Triangle (2, 61, 173)	Triangle (2, 61, 173)	Triangle (2, 61, 173)	Triangle (2, 61, 173)
Number of premises in MR given number of infected premises (regression slope)	Normal (10.91, 0.186)	Normal (17.75, 1.15)	Normal (24.83, 1.93)	Normal (35.82, 2.17)	Normal (42.5, 3.16)
% overlap given number of infected premises (regression slope)	Normal (0.0175, 0.0009)	Normal (0.0213, 0.0009)	Normal (0.023, 0.001)	Normal (0.0224, 0.002)	Normal (0.0267, 0.002)
% overlap given number of infected premises (regression intercept)	Normal (−0.0244, 0.012)	Normal (−0.0297, 0.015)	Normal (−0.0174, 0.015)	Normal (−0.005, 0.028)	Normal (0.002, 0.033)
Maximum overlap	Triangle (0.75, 0.85, 0.95)	Triangle (0.75, 0.85, 0.95)	Triangle (0.75, 0.85, 0.95)	Triangle (0.75, 0.85, 0.95)	Triangle (0.75, 0.85, 0.95)

ArcGIS (version 10.3, ESRI, Redlands, CA, USA) was used to map the infected zones and movement restriction zones for each of the index premises at the first time step of the eight most-likely CSF outbreak scenarios. The premises that were within-movement restriction zones were identified and counted. Two linear regression equations were developed based on these data to estimate the number of swine premises that would fall under movement restrictions during an outbreak (Table 3). As shown in Figure 1B, some premises might fall under multiple movement restriction zones (i.e., overlap) in a multiple-site outbreak. The percentage of overlapped swine premises was estimated for a given number of infected premises to approximate the number of unique swine premises. If the overlap percentage was greater than or equal to a predetermined maximum overlap percentage, the maximum percentage of overlap (*maximum overlap*, in Table 3) was used. The ED was estimated from the CSF spread model described in Section “Simulation of CSF Spread.” Unlike multiple-site outbreak type, the ED estimates from the single-site outbreak type had a bimodal distribution (denoted by *ED low and ED high* in Table 3). The probability distribution of ED in the single-site outbreak scenario was represented by two trianglular (minimum, most likely, maximum) distributions along with a Bernoulli (probability of event) distribution, which represented the probability of occurrence of either of the ED (low or high) distributions (Table 3).

Adverse animal welfare consequences of movement restriction investigated in this study included overcrowding and feed interruption. When the total weight of pigs on premises exceeded 100–115% of the maximum capacity of the premises, the condition was referred to as overcrowding. The maximum capacity of swine premises was calculated as the total weight of pigs on premises at the age of harvest. Feed interruption was modeled as a farmer’s decision to discontinue feed supply, followed by the euthanasia of the pigs. The decision of feed discontinuity was dependent on the ED estimated at the onset of an outbreak, the duration between the initial age of the pigs at the start of an outbreak and the harvest age, and the progress (number of days) of an outbreak; longer durations of these factors resulted in the farmers’ being more likely to discontinue the feed supply. We developed a model to estimate the time when overcrowding or feed interruption emerged (TWC) in our previous study (10). Probability distributions for TWC were derived from those estimates.

The number of daily newly infected swine premises was estimated from the CSF spread model using the NAADSM (see Simulation of CSF Spread). The number of unique swine premises under movement restrictions was then estimated as a function of the number of infected premises using the regression equations (Table 3). The model algorithms compared TWC with ED to flag when TWC < ED, indicating that the premises would experience adverse animal welfare outcomes before the outbreak ended. A total of 26 different simulations were run using @Risk software (Palisade Corporation, Ithaca, NY, USA) with 100,000 iterations each. Latin hypercube sampling with a Mersenne Twister generator of randomly selected initial seed was used.

### Sensitivity Analysis

For the CSF spread model, direct contact rate and probability of CSF transmission were chosen for the sensitivity analyses because of their major roles in disease spread and also the inconsistent estimates across studies (18, 20, 26, 30). A 25% change in the values of these parameters was used in the analyses to evaluate their influences on the estimates of ED and the percentage of infected premises. A parameter was considered influential if the median ED changed by 14 days or the median percentage of infected premises changed by 25% compared with the baseline.

For the animal welfare risk assessment model, the Spearman correlation was performed on all input parameters to assess their correlation with the outcome (i.e., the number of premises that would experience animal welfare outcomes). The parameters that resulted in a correlation coefficient ≥0.3 were included in the further sensitivity analysis. A 25% change in the values of the selected parameters was used in the analyses to evaluate their influences on the estimates of the median percentage of premises that would experience animal welfare outcomes. An input parameter was considered as influential if the outcome changed by 25% compared with the baseline.

### Statistical Analysis

Kruskal–Wallis tests were used to compare among the movement restriction zone sizes. Dunn’s tests, adjusted for multiple comparisons, were used to compare between 7-km zone size and the other zone sizes after a significant Kruskal–Wallis test. Statistical significance is defined as *P* ≤ 0.05.

## Results

A total of 71 infected zones and movement restriction zones were identified and mapped for the zone sizes of 3, 5, 7, 9, and 11 km in the 8 selected CSF outbreak scenarios. The median number of swine premises in the infected zone (3-km radius of index premises) at the onset of the outbreak was 6 (range: 5–16) and 94 (range: 16–145) in the single-site and multiple-site outbreak type, respectively. Details of the number of unique swine premises in the various movement restriction zone sizes at the onset of an outbreak are presented in Table 4.

**Table 4:** **Median (range) number of swine premises in the movement restriction zone sizes of 3, 5, 7, 9, and 11 km at the onset of single-site and multiple-site CSF outbreak scenarios in Indiana**.

Outbreak scenarios	Movement restriction zone sizes				
	3 km	5 km	7 km	9 km	11 km
Single site	22 (10–49)	42 (16–75)	67 (26–96)	93 (58–132)	122 (67–174)
Multiple site	207 (45–280)	353 (75–428)	521 (137–559)	735 (205–824)	876 (268–979)

### Epidemic Duration

**Figure 2:**
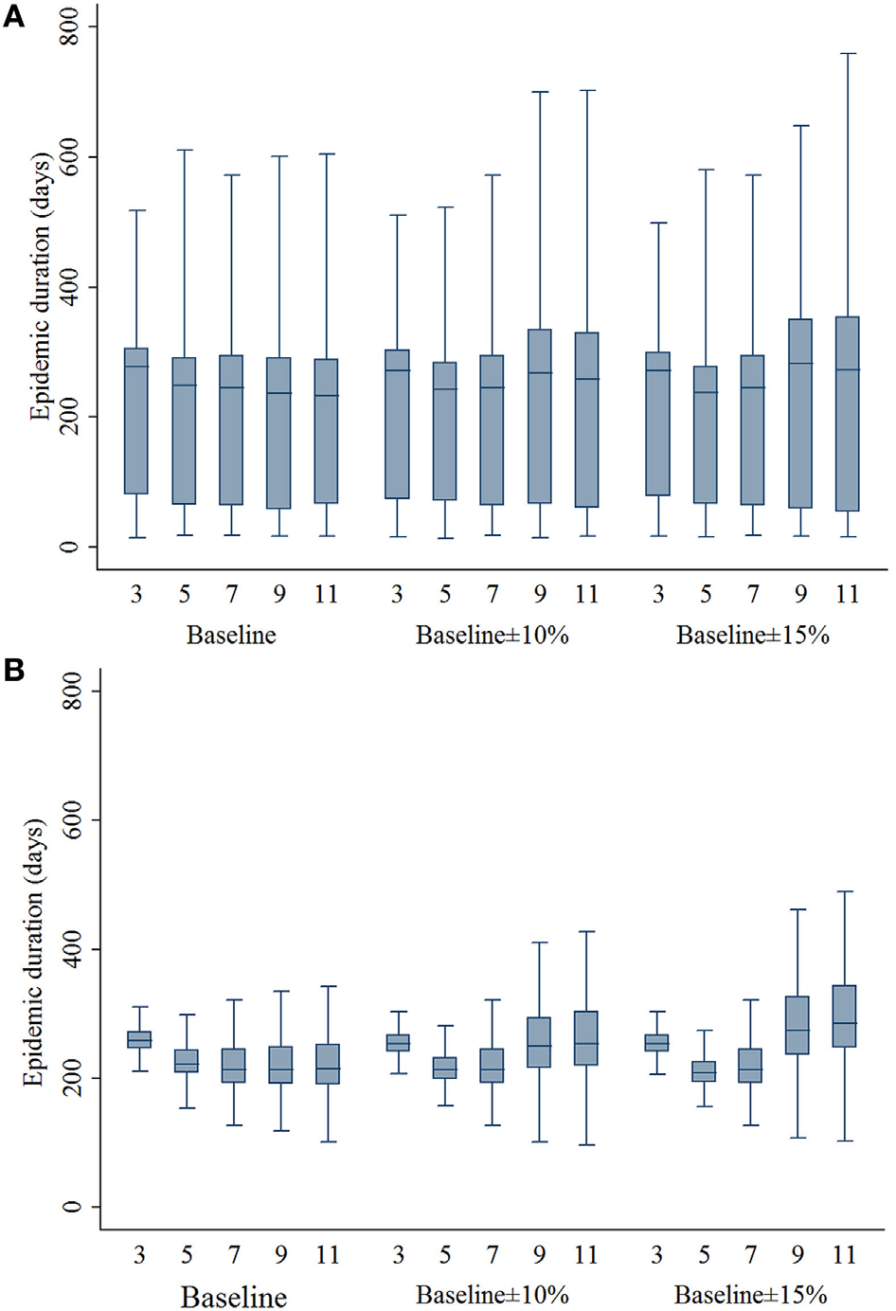
**Boxplots of epidemic duration estimates resulting from the movement restriction zone sizes of 3, 5, 7, 9, and 11 km by different compliance assumptions (baseline, baseline ± 10%, and baseline ± 15%) in (A) single-site and (B) multiple-site classical swine fever outbreak types in Indiana**. The lower edge, central line, and upper edge of box represent 25th percentile, median, and 75th percentile, respectively. The whiskers represent the data range or ±1.5 × interquartile range from the 25th and 75th percentiles.

The estimates of ED resulting from different movement restriction zone sizes are presented in Figure 2. In the baseline compliance simulations, 3- and 5-km zone sizes resulted in a significantly longer ED compared with the 7-km zone size in both outbreak types (*P*’s < 0.001). In the baseline ±10% simulations, EDs resulting from 3-, 9-, and 11-km zone sizes were longer compared with the 7-km zone size (*P*’s < 0.001), whereas the EDs resulting from 5- and 7-km zone sizes were not different in both outbreak types. In the baseline ± 15% simulations, 3-, 9-, and 11-km zone sizes resulted in a significantly longer ED (*P*’s < 0.001), whereas the 5-km zone size resulted in a shorter ED (*P* = 0.036) compared with the 7-km zone size.

### Infected and Preemptively Depopulated Premises

**Figure 3:**
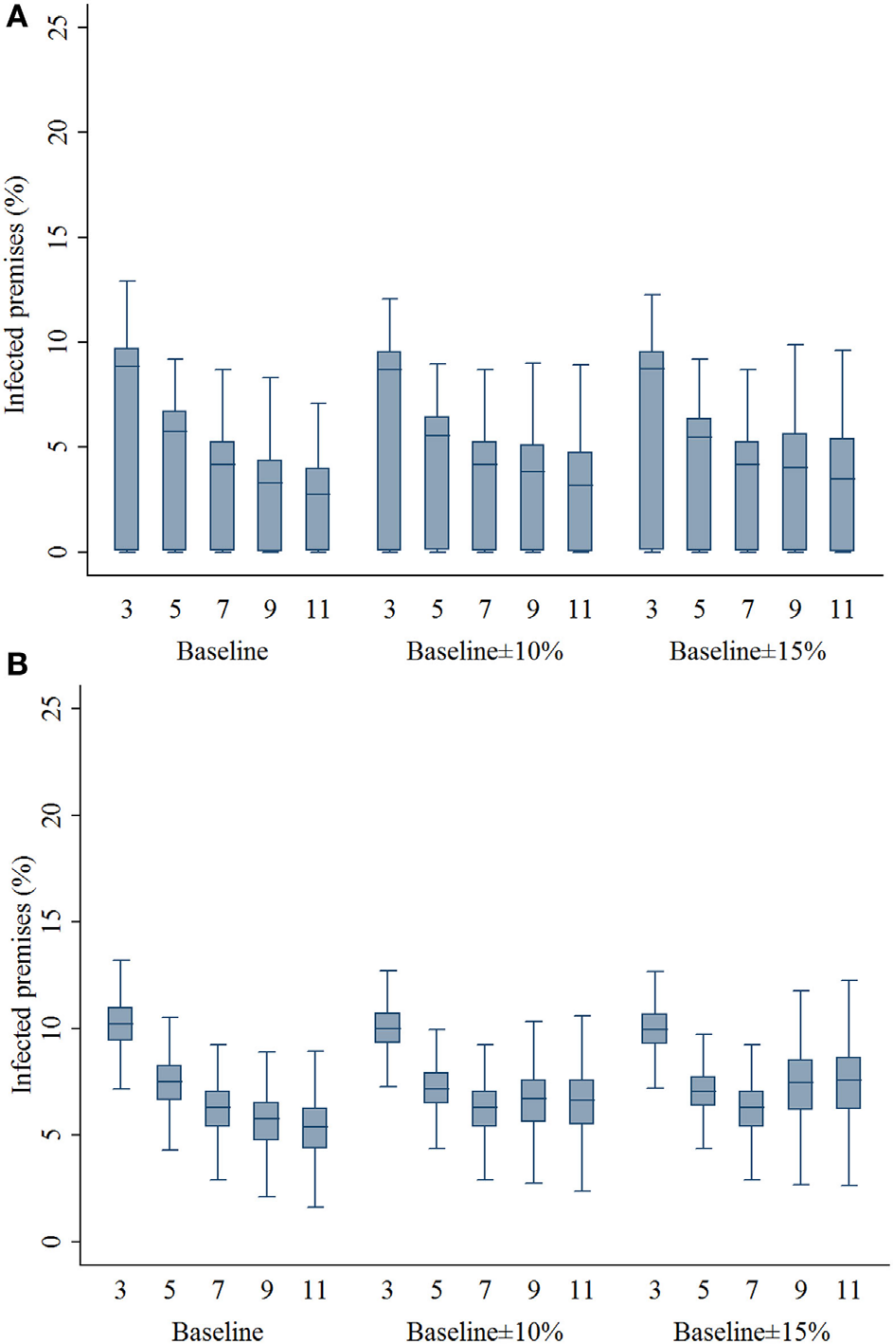
**Box plots of percentage of infected swine premises resulting from the movement restriction zone sizes of 3, 5, 7, 9, and 11 km by different compliance assumptions (baseline, baseline ± 10%, and baseline ± 15%) in (A) single-site and (B) multiple-site classical swine fever outbreak types in Indiana**. The lower edge, central line, and upper edge of box represent 25th percentile, median, and 75th percentile, respectively. The whiskers represent the data range or ±1.5 × interquartile range from the 25th and 75th percentiles.

**Figure 4:**
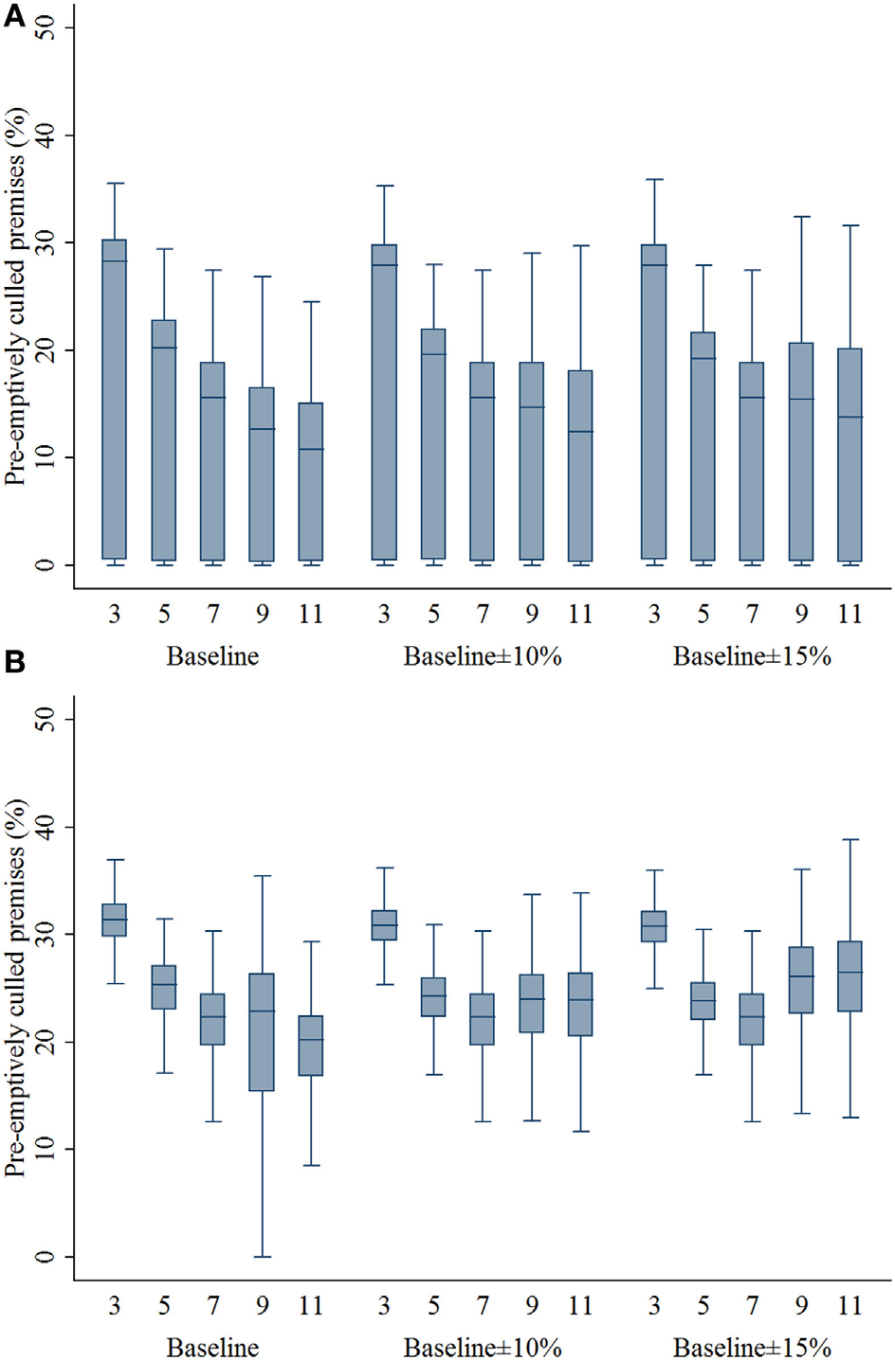
**Box plots of percentage of preemptively culled swine premises resulting from the movement restriction zone sizes of 3, 5, 7, 9, and 11 km by different compliance assumptions (baseline, baseline ± 10%, and baseline ± 15%) in (A) single-site and (B) multiple-site classical swine fever outbreak scenarios in Indiana**. The lower edge, central line, and upper edge of box represent 25th percentile, median, and 75th percentile, respectively. The whiskers represent the data range or ±1.5 × interquartile range from the 25th and 75th percentiles.

Compared with the 7-km zone size, the 3- and 5-km zone sizes resulted in a higher percentage of infected swine premises in both outbreak types (*P*’s < 0.001; Figures 3A,B). With the assumptions of lower compliance levels, the zone sizes of 9 and 11 resulted in a higher percentage of infected premises in the multiple-site outbreak significantly (*P*’s < 0.001). In the single-site outbreak, the percentage of infected premises resulted from 9-km zone size was not different from that of 7-km zone size (*P* = 0.537). Results of the percentage of infected premises are presented in Figure 3. As with the infected premises estimates, a similar pattern was found in the percentage of preemptively culled swine premises (Figure 4).

### Adverse Animal Welfare Outcomes

**Figure 5:**
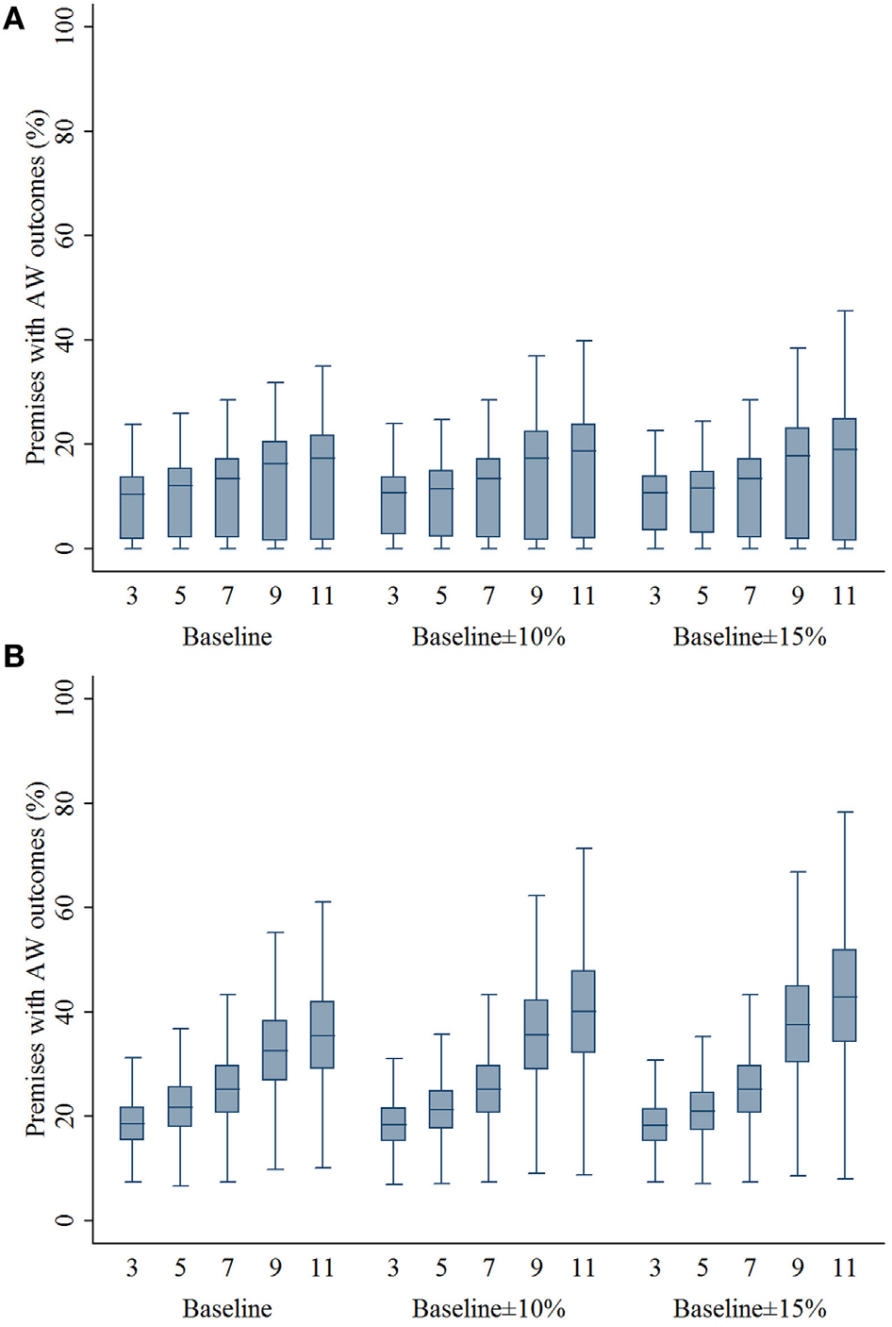
**Box plots of percentage of swine premises experiencing adverse animal welfare outcomes resulting from the movement restriction zone sizes of 3, 5, 7, 9, and 11 km by different compliance assumptions (baseline, baseline ± 10%, and baseline ± 15%) in (A) single-site and (B) multiple-site classical swine fever outbreak types in Indiana**. The lower edge, central line, and upper edge of box represent 25th percentile, median, and 75th percentile, respectively. The whiskers represent the data range or ±1.5 × interquartile range from the 25th and 75th percentiles.

The percentage of swine premises that would experience adverse animal welfare outcomes due to movement restriction was positively associated with the zone size regardless of compliance levels and outbreak types (Figure 5). The median percentage of swine premises that would experience adverse animal welfare outcomes was 10–27% lower comparing 3- and 5-km zone sizes with 7-km zone size (*P*’s < 0.001).

### Composition of Depopulated Swine Premises

**Figure 6:**
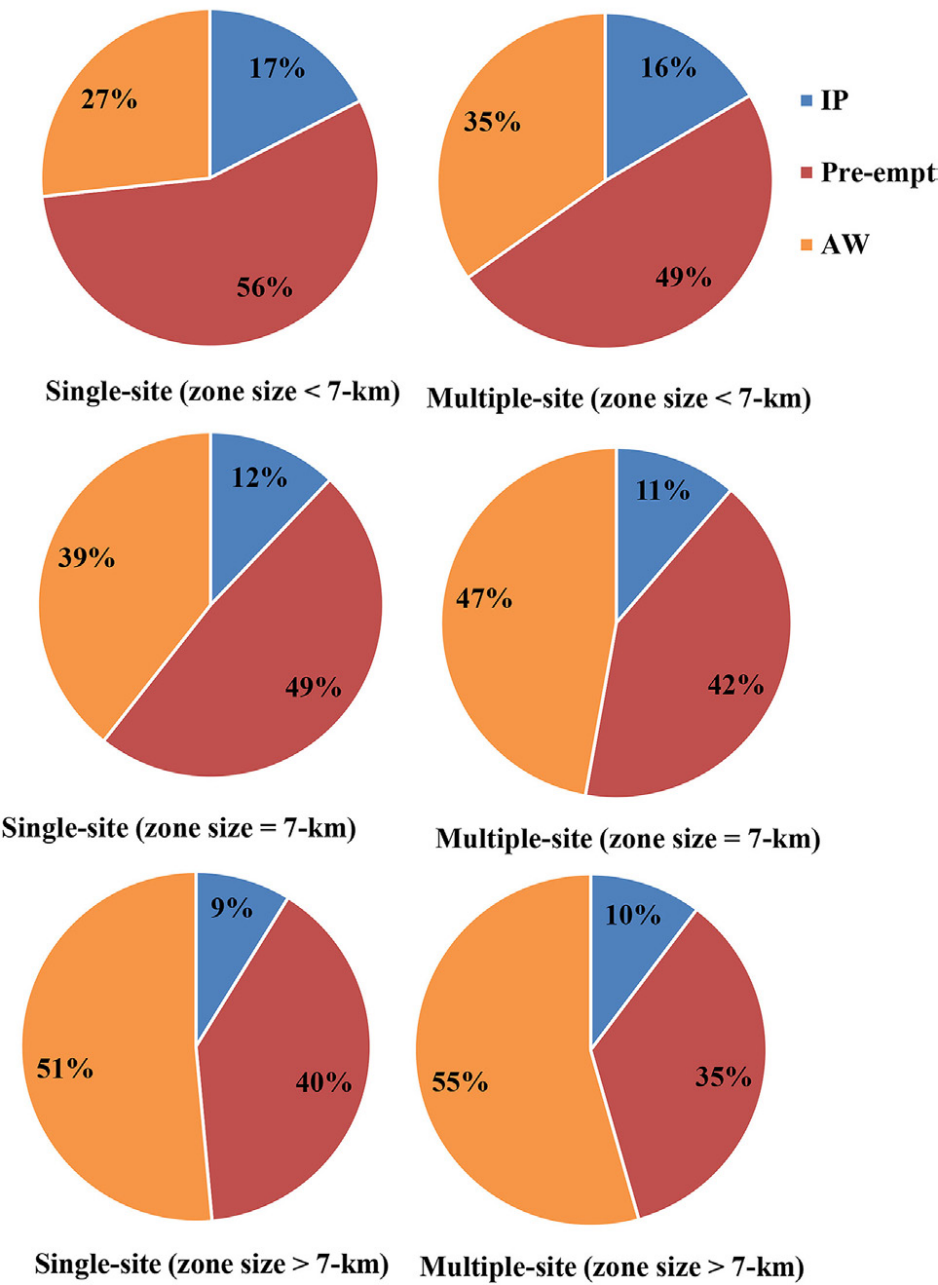
**Pie chart represents the percentage of total depopulation of swine premises due to infection (IP), pre-emptive culling (Pre-empt), and adverse animal welfare outcomes (AW) for small zone sizes (3 and 5 km), 7-km zone size, and big zone sizes (9 and 11 km) by outbreak types (single site and multiple site)**.

The results of composition of depopulation (due to infection, preemptive culling, and animal welfare outcomes) are presented in Figure 6. In an event of CSF outbreak in the United States, infected and contact premises and premises experiencing animal welfare outcomes are all subject to depopulation. Among the small zone sizes (3 and 5 km), majority of the swine premises to be depopulated were due to preemptive culling (single-site outbreak: 56%, multiple-site outbreak: 49%), whereas among the large zone sizes (9 and 11 km), the majority to be depopulated were due to animal welfare outcomes (single-site outbreak: 51%, multiple-site outbreak: 55%). For the 7-km zone size, 48 and 42% of the swine premises were preemptively depopulated and 39 and 47% were depopulated due to animal welfare outcomes in the single-site and multiple-site outbreak, respectively.

### Sensitivity Analysis

The results of the sensitivity analysis for CSF spread model showed that direct contact rate and probability of infection transfer were influential on the percentage of infected premises estimations in the single-site outbreak. In the multiple-site outbreak, a 25% increase in direct contact rate or probability of infection transfer altered the estimate of median percentage of infected premises by 26–65% across different zone sizes. They showed a greater influence on the percentage of infected premises estimate for 3-, 9-, and 11-km zone sizes.

Direct contact rate and probability of infection transfer were also influential on ED estimations for all zone sizes in the multiple-site outbreak. In the single-site outbreak, a 25% increase in direct contact rate and probability of infection transfer altered the ED estimate by 22–45 days.

For the animal welfare risk assessment model, maximum overlap percentage (*maximum overlap*; Table 3) and probability of ED low (*Prob_ED_low*; Table 3) showed a correlation with the number of premises experiencing animal welfare outcome estimate (i.e., Spearman’s correlation coefficient ≥0.3) and were included in the further sensitivity analysis. The results of the sensitivity analysis showed that a change in *maximum overlap* by 0.05 altered the outcome estimate up to 27% across different zone sizes in the multiple-site outbreak. In the single-site outbreak, a decrease in the *maximum overlap* by 0.05 did not affect the outcome estimate, whereas an increase by 0.05 reduced the number of affected swine premises by 29–41% across the investigated zone sizes. *Prob_ED_low* was changed to Bernoulli (0.1) and Bernoulli (0.4) from the baseline distributions in the sensitivity analysis. These changes in *Prob_ED_low* did not influence the median percentage of swine premises that experienced animal welfare outcomes estimate.

## Discussion

We evaluated the impact of various movement restriction zone sizes on CSF outbreak control compared with the recommended zone size of 7 km using Indiana swine premises data. The study findings provide evidence-based foundation for decision makers to determine the optimal movement restriction zone size for CSF outbreak controls.

The investigated movement restriction zone sizes showed variable effects on the ED estimates depending on the outbreak types (e.g., single-site and multiple-site outbreaks) and compliance levels. Across the outbreak types, the differences in ED estimates among zone sizes were slightly greater in multiple-site outbreak than in single-site outbreak. The results also suggested that ED did not always decrease with an increase in the zone size. This was contradictory to the presumption of the monotonically negative relationship between movement restriction zone size and ED (6, 31).

Similarly, it was found that the relationship between movement restriction zone size and the percentage of swine premises to be depopulated (infected premises, preemptively culled premises, and premises with animal welfare outcomes) was not necessarily directional. Both compliance level and outbreak type influenced the effects of movement restriction zone size on the percentage of swine premises to be depopulated. However, the 3-km zone size consistently increased the number of total depopulated swine premises across all simulated scenarios. A closer look at the simulation results revealed that a 3-km zone size resulted in the largest number of daily new infections compared with other zone sizes. With a constant compliance level (i.e., baseline compliance simulation), 5-, 9-, and 11-km zone sizes resulted in the similar percentage of premises to be depopulated. Assumption of a low compliance level for a larger zone size led to a higher percentage of depopulation for zone sizes of 9 and 11 km; a greater difference was found in the multiple-site outbreak compared with single-site outbreak. The finding that the differences in outcome estimates tended to be lager in the multiple-site outbreak might be explained by that a multiple-site outbreak, on average, resulted in more new infections. Overall, 7-km zone size was found to result in the lowest percentage of premises to be depopulated, whereas the 5-km zone size showed comparable results in the baseline compliance simulation and performed better with the assumption of a higher compliance level in the multiple site. Having fewer premises that need to be depopulated during an outbreak may greatly reduce the overall burden (e.g., resources for euthanasia, carcass disposal, and transport) for outbreak controls (32, 33). During the 1997–1998 CSF outbreaks in the Netherlands, seven million pigs were euthanized to alleviate adverse animal welfare outcomes, which competed for the limited resources going toward euthanizing infected pigs, carcass disposal, biosecurity, and disinfection. Consequently, the epidemic was prolonged, which might have caused additional animal welfare issues in pigs and economic losses (4, 34). A similar challenge of insufficient resources for euthanasia, carcass transport, and disposal was observed in the highly pathogenic avian influenza outbreaks in the United States in 2014, which also hindered the timely management of outbreaks (35).

Our models showed that preemptive culling and euthanasia due to adverse animal welfare outcomes were the major contributors to the total depopulation, whereas the infected premises contributed the least (Figure 6). This finding agrees with the reports from historical CSF outbreaks (3). Our results further indicated that the composition of depopulation was dependent on movement restrictions zone size. In the small zone size group, the majority of swine premises were euthanized due to preemptive culling, whereas in the large zone size group, adverse animal welfare outcomes contributed the most. In the historical outbreaks, euthanasia of pigs due to adverse animal welfare outcomes contributed 64% to total pigs euthanized (6, 8). The study model showed that movement restriction zone size of 5 km could result in 20% fewer premises to be euthanized due to adverse animal welfare outcomes compared with the zone size of 11 km. These findings underscore the significance of animal welfare problems if a bigger movement restriction zone size is designated. Our assessment of different outbreak-related outcomes provide more comprehensive evidence to assist decision makers and disease control authorities in designating an optimal movement restriction zone size for CSF outbreak control. Awareness of swine producers toward these crucial outcomes might also help them be better prepared to prevent devastating consequences in an event of CSF outbreak.

Overall, the 7-km movement restriction zone size had the best performance in CSF outbreak control based on our models. It resulted in the shortest ED and the lowest percentage of swine premises to be depopulated compared with the other investigated zone sizes. A shorter ED will reduce the international trade ban period, which will lead to a quicker recovery of business. Furthermore, implementing a 7-km zone size may reduce the demand of limited resources for outbreak controls, which is crucial for smooth and efficient outbreak management. Under certain circumstances, such as in an event of multiple-site CSF outbreak, a 5-km zone size might be as effective as a 7-km zone size in controlling the outbreak in Indiana; particularly if a higher compliance level can be achieved.

Movement restrictions will inevitably also affect regular movements of pigs outside the designated control zones. Further investigation by including the movement data outside the control zones is warranted to more comprehensively evaluate the impacts of movement restriction zone size. During a CSF outbreak, the interstate network of swine movement might result in a rapid spread of CSF beyond Indiana. Therefore, including the other partnering swine producing states of Indiana in future studies might help achieve better insight about the role of movement restriction zone size in controlling a multistate CSF outbreak. The extrapolation of the study findings to the swine industry of other US states and countries should be done with due consideration of the assumptions in the model. The results and simulation outputs from this study could be used in the cost–benefit analysis of movement restriction in controlling a CSF outbreak.

## Conclusion

The effectiveness of movement restriction zone size in controlling a CSF outbreak were dependent on various factors, such as outbreak type, compliance with movement restriction, and outcome measure of interest. Our findings indicated that a 7-km zone size was the most effective in reducing the ED and percentage of swine premises that need to be depopulated compared with other investigated zone sizes. The zone size of 5 km was comparable with the 7-km zone size particularly in the multiple-site outbreak with an assumption of a higher compliance level.

## Author Contributions

SY: conceived and designed the study, performed data collection, simulation, data analysis, and manuscript writing. NOW: contributed in data analysis, manuscript writing, and review. DL: contributed in manuscript writing and review. CC: contributed in manuscript writing and review. H-YW: contributed in study design, data analysis, manuscript writing, and review.

## Conflict of Interest Statement

The authors declare that the research was conducted in the absence of any commercial or financial relationships that could be construed as a potential conflict of interest.
